# Improving children and adolescents’ quality of life, personal growth, well-being, and safety through health-behavioral education: a pre-post intervention study

**DOI:** 10.3389/fpubh.2025.1527268

**Published:** 2025-04-17

**Authors:** Czarina Leung, Tim Leung, Daphne Lau, Rachel Ng, Shahira Fatin, Shelly Chutke, Cayla Pui, Jing Xi Dai, Josephine Yu, Jason Jia

**Affiliations:** Be Priceless, Hong Kong, Hong Kong SAR, China

**Keywords:** child, adolescent, health behavior, health education, behavioral intervention, risk reduction, health and well-being, quality of life

## Abstract

**Introduction:**

Hong Kong children and adolescents are living with alarming deterioration of mental health and escalating risks of violence and self-harm. Self-value, Empower, Educate, and Protect from Dangers (SEED) health-behavioral education provided to children, caregivers, and care service providers by Be Priceless is the only Hong Kong program that integrates safety with mental, physical, and social well-being.

**Methods:**

526 children aged 6 to 17 years were recruited into a pre-post intervention study from May 2021 to September 2024. The impact of our SEED health education intervention was evaluated on four outcome measures: health-related quality of life, emotional regulation, well-being/resilience, and safety.

**Results:**

Relative to pre-course baseline, all four measures showed significant improvements after intervention which persisted for 6 months, with large effect sizes (Cohen’s d = 0.66–0.92, all *p* < 0.001), and especially for those who were ethnic minorities or with special education needs.

**Discussion:**

These results provide evidence that the SEED health education of Be Priceless is an efficacious intervention that improves children’s well-being and safety, while promoting health equity and inclusivity in Hong Kong.

## 1 Introduction

Childhood and adolescence are critical periods associated with rapid changes in physical-socio-emotional developments, learning, and interpersonal relationships. These changes could influence their short-term and long-term health, including in physical, mental, and social well-being, and safety (1, 2). Thus, it is of vital importance to implement effective risk-informed education during childhood and adolescence that empowers them with attitudes, behaviors, and capacities that strengthen their overall health.

Similar to many other places, Hong Kong’s children and adolescents are facing alarming deterioration of mental health and escalating risk of violence and self-harm (3–10). The Hong Kong Happiness Index 2022 dropped to 6.77 (out of 10) from 6.85 in 2021 (3). Academic pressure, low parental effective communication and respect of opinion were key risk factors. 40–60% of primary and secondary school students have depressive or anxiety symptoms (4, 5). Primary and secondary school children suicide rates has doubled since 2018 (11).

Those who are most vulnerable to physical, mental, and social health risks lack the necessary service and parental training. Case interview conducted by Student Suicide and Help-Seeking Attitudes by Hong Kong Christian Service in 2023 showed that when students face negative emotions, only about 10% would seek help from caregiver due to “worrying about being exposed or ridiculed” (30.0%), “fear of being criticized or questioned” (16.7%), and “not being understood” (14.0%) (6).

Against Child Abuse has record 187 suspected child abuse cases in different levels (56% of physical abuse, 9% of sexual abuse, 16% of psychological abuse) and nearly 70% of suspected abusers were family members. It causes far-reaching impact on children’s well-being in lack of sense of security, low self-confidence and self-image, doubts about their own values, low level of trust in people and even obstacles in interpersonal relationships (7).

Children living with special education needs (SEN) or in poverty face disproportionate bio-psychosocial health risks, including mental health issues, violence and lower health status. The risks are particularly high for ethnic minority (ethnically non-Chinese) children (12). Such health disadvantages accumulate throughout life course, and contribute to worsened health and the social determinants of health, including their health behaviors, access to health education, academic education, supportive social networks and more (13–15). Addressing these needs are especially urgently warranted, as they interrupt the cascades of mental health problems, inequities, and loss of opportunities to flourish, to reach their full potential over the course of their lives and to contribute to society in their unique ways.

Currently, there are no educational programs in Hong Kong that build the behaviors and preparedness of children for safety and well-being in the context of mental health, violence, or other health crises (16). There is little evidence of interventions that improve health-related quality of life or well-being of children and families.

### 1.1 SEED health behavioral education program development

The SEED (Self-value, Empower, Educate, and Protect from Dangers) health behavioral education provided by Be Priceless is the only Hong Kong program that integrates safety with well-being of the mind, body, and relationships for children (17). This program was developed to enhance the well-being and safety of children, youth, and caregivers, inclusive of those who are from at-risk backgrounds (e.g., from SEN, low-income, minority ethnic, migrant, refugee or asylum seeking families). Overall over 70 professionals from more than 10 fields and over 150 diverse community members (including children and caregivers) participated in the development of the course.

To understand the situations, unmet needs, and trans-sectoral best practices for improving children’s mental-physical-social health, a consultative process was conducted with diverse front-line service professionals taking care of children’s health, well-being, or safety. This included social workers, school counselors, educators, doctors, nurses, child protection professionals, anti-human trafficking experts, lawyers, and more. These insights along with research of evidence-based practices (18–28) and reputable frameworks (including WHO, United Nations, CASEL) were integrated with the coordinating authors’ expertise in medicine, public health, and health education to create the draft version of the SEED Educational curriculum topics and the draft content of some of the educational animations and comic books intended to provide awareness and knowledge of key concepts, mindsets, and actions for self-efficacy, mental-physical-social health, well-being and safety (29–38).

A situational analysis was conducted with 105 caregivers of children and 28 staff of non-profit organization provided their perspectives on the unmet needs and challenges for children’s well-being and safety in focus group semi-structured interviews. This formed the basis of contextualizing the educational program that aimed to address these unmet needs (39).

In alignment of the insights from the situational analysis and latest best practices in diverse relevant fields, Be Priceless formed a trans-sectoral team to design and develop the SEED curriculum, educational content (including class plans, teaching slides, interactive activities), improved the educational tools (e.g., animations, comic books) as well as its delivery. This team consists of professionals with expertise in providing training to children. They include teachers, counselors, play and art therapists, mindfulness coaches, social workers (with mental health expertise), doctors, a nurse, and others.

Components of the educational content were internally and externally reviewed relevant experts. For example the module on personal safety and reducing the risks of violence was reviewed by staff from Save the Children HK, the module on stopping exploitation was reviewed by experts in the International Organization of Migration Hong Kong SAR, the module on mental health and well-being was reviewed by external mental health professionals, and the module to staying safe from respiratory infection was reviewed by senior intensive care doctors in HK as well as public health and risk communications experts in major international organizations. Their comments were addressed by the trans-sectoral team.

During these processes, diverse community members participated. For example, multicultural adolescents, children, and caregivers took part in co-developing some of the animation scripts and performing the voiceovers. Some developed with the team the interactive games to bring to life the concepts taught, reviewed the educational materials, translated, and collaborated with our team in other ways.

The impact evaluation was developed by another team, all but two of the members had been involved in the course creation (Please see the “Measures” section below for details).

The children’s SEED Course and the impact evaluation was piloted with 7 adults who had not been involved in the course development and subsequently revised according to their feedback. The next pilot course was received by a group of 5 children aged 8 to 14 years, and then further improved. This course then rolled to be provided for a group of 49 children with the mean age 10.0 +/− 2.3 years. A longer focus group and interview was conducted at the end of the course in order to understand the learning outcomes better. From their feedback and evaluation, the course and the evaluation was further improved, made more concise, and more engaging. This improved SEED Course and version two of the impact assessment are discussed in this paper.

### 1.2 The study

This study aims to evaluate the impact of the SEED course of Be Priceless, a transdisciplinary health behavioral-educational intervention, on children and adolescent learners’ health and behavioral outcomes in the domains of quality of life, personal growth, well-being, and safety.

## 2 Materials and methods

### 2.1 Participants and study design

A pre-post intervention study design was used to evaluate the impact of courses on the children participants.

Participants were recruited either from the general public in Hong Kong (e.g., they voluntarily sign up through the Be Priceless website, promoted by social media posts, posters, brochures, or other community members) or via partner schools or non-profit organizations that served communities facing higher risks, including low-resourced, minority ethnic, migrants, asylum seekers, refugees, or other vulnerable groups.

The inclusion criteria were children 6–17 years old. All participants must live in or go to school in Hong Kong.

The exclusion criteria were (1) children’s guardians who could not or did not give consent, and (2) children who were unable to communicate in verbal or written format that can be understood by the researchers (English, Cantonese, Mandarin) or by our available translators (Hindi, Nepali, Urdu, Indonesian Bahasa).

From May of 2021 to September of 2024, A total of 629 children completed the Pre-course survey after being their caregivers signed up for the SEED Course. 103 children did not complete the course. Their reasons for dropping out included personal reasons (e.g., health reasons, language barrier; 73%), time clashed with other activities (26%), and others (1%). 526 children and youth aged 6–17 years who completed the SEED Course participated in the study. All participants (or the guardians of participants under the age of 18 years) have provided written informed consent. Chinese and English were the main languages used in the assessments and courses, but where needed translation was provided, e.g., Bahasa-Indonesian, Urdu, Nepali, Hindi translation for ethnic minority children.

### 2.2 Procedures

The SEED Course is an education behavioral program that aims to cultivate children’s behaviors for strengthening their growth, well-being and safety. The pedagogical strategy is learner-centered, age-appropriate, educational and behavioral intervention that supports a whole-child health, rights-based learning, and multi-hazard risk reduction. The Course empowers learners’ attitudes, behaviors, capacities, and collaborations for four key areas: (1) personal growth (including self-value, socio-emotional learning), (2) well-being of the mind and body, (3) safety (preventing of, preparing for, responding to, and recovering from diverse risks including (i) personal mental and physical health problems, (ii) interpersonal violence and exploitations both online and in-person, (iii) community disorders such as inequity, discrimination, and misinformation; and (iv) environmental risks such as infectious outbreaks, extreme weather events, environmental degradation and climate change related risks). The course consists of eight modules:

Module (A) I am a growing seed (self-value, self-agency, inner growth),

Module (B) Positive ways of seeing myself (growth mindset, resilience),

Module (C) Risk reduction (understanding diverse risks commonly affecting children including extreme weather events and climate change; risk awareness, assess own risks; practice risk prevention, preparedness, response and recovery),

Module (D) The pause (self-awareness, mindfulness),

Module (E) My mind and body belong to me (personal boundaries, unsafe behaviors, violence risk awareness; practice risk prevention, preparedness, response and recovery).

Module (F) Mental Health and well-being, Module (G) Stay away from respiratory infections, and Module (H) Stop exploitation (including understanding online and human trafficking risks; risk prevention, preparedness, response and recovery).

The implementation is adapted to the child learners’ needs, context, and capacity. For example, the scenarios and solutions will be made to reflect the situations that children of that specific age group and community may face. The educators are fully trained in delivering the course and upholding the child safeguarding and protection protocols. The trainers consist of multidisciplinary expertise, including doctor, nurse, counselors, teachers. There are always at least two adults in the classroom who are trained in the intervention’s operational and integrity protocols.

Children engage through gameful learning that includes experiments, role-playing, storytelling, discussions, practicing emotional regulation and self-value during class challenges, using teamwork and healthy decision-making to develop solutions for inequalities, and rehearsing risks assessment and safety actions such as asking for help for big problems such as mental disorders and violence. We provide learning tools for in-class and for home practices, e.g., via personalizable exercises, journal and safety plan. The educators engage the learners through collaborations on establishing classroom culture and use of fun learning tools, such as animations, games, songs, and dance moves. Some of these resources are available for public use on www.bepriceless.org.

The course guides children to understand that there are different layers of influence on their growth, well-being and safety including intrapersonal (their own mind and body), interpersonal (including relationships with their caregivers and others close to them), their community (including their school), and their environments.

The course usually lasts for 12-h for each group of students. For children who are recruited from the general public, the course often runs a 6 sessions (2 h/session) program. For children who are recruited via partners (schools or non-profit organizations), the course often runs as an intensive 3 half-day program, or a 6 sessions (2 h/session) program. The specific schedule is adapted to the needs and availability of the children.

#### 2.2.1 Educators

To ensure equal and high quality of delivery of the educational program, measures were implemented in (i) educator recruitment, (ii) standardized course content, (iii) rigorous educator training and supervision.

- *Educator recruitment:* to provide the courses for this cohort of children, 5 educators were involved. Their backgrounds included psychology, early childhood education, communication, medicine (nurse), music and Chinese language
- *Standardized course content: t*he educational content (classroom agreements, slides, interactive activities with integrated behavioral interventions), tools (including rap, songs, dance, animations) and home practices (safety plan, growth journals, comic books) were already fully developed prior to the study. The teaching slides have scripts and detailed descriptions of activities for educators to follow. The detailed descriptions of educational activities included the timing, the set up of the environment, and written guides on how the educator may engage the child learners for depth of behavioral change.
- *Rigorous training and supervision:* Each educator received around 100 h of training, including reviewing online training materials; attending or watching videos of senior educators’ (trainers) classes; coordinated classes taught by senior educators; mock teaching of each of the SEED lesson to the trainers with feedback on their delivery, class management, and safety protocol implementations; co-teaching with senior educators with feedback after each class; and finally lead teaching classes with a senior educators’ supervision.

### 2.3 Measures

A health behavioral survey was developed by a multidisciplinary team from medicine, psychology, education, research, non-profit program evaluation, and public health to ensure a holistic approach to assess the impact of the intervention on the participants’ health-related quality of life, emotional regulation, well-being, and safety.

The team conducted rigorous literature review to identify evaluation instruments of health, personal growth, well-being, and safety (including against violence) for children 6–17 year-old or caregivers of children. Then based on their relevance to the topics covered by the SEED Course and the suitability for the learners (taking into account their age, literacy, primary language, and locality) together they decided on selecting several instruments. No appropriate local tools were found for children’s well-being and personal safety. Therefore, for the pilot version of the survey, the team selected internationally validated tools, and plan to co-develop through a participatory approach with diverse local children and caregivers a Community Score Card for measuring children’s flourishing, personal growth, well-being and safety from their perspective (to be published later and is integrated into version 2 and 3). In this paper, we are describing the results from version 2 of the survey implementation. Version 3’s data collection is in progress.

The tool for measuring the health and health behaviors had 24 questions measuring: health-related quality of life (KIDSCREEN-27) (40), emotional regulation (Panorama) (41), well-being/resilience and safety (from our Community Score Card) (42).

The internal validity the Cronbach’s alphas for the domains were as follows: Health-related quality of life (11 items) pre-course and post-course are 0.794 and 0.874 respectively, emotional regulation (5 items) 0.756 and 0.818, well-being/resilience (5 items) 0.740 and 0.844, Safety (3 items) 0.303 and 0.526. Answers were most inconsistent in the one safety question was flipped with 1 being strongly agree and 5 being strongly disagree - if this question was deleted the Cronbach’s alpha would increase to 0.394 and 0.591.

All participants completed the survey before (“Pre-course”) and at the end of the course (“Post-course”). For those who attended follow-up (“Follow-up,” usually around 6 months after the course), the survey was repeated.

#### 2.3.1 Community score card

To co-create a localized set of impact measures of children’s health behaviors, in a separate study we performed a participatory project with 151 diverse children and 217 caregivers from Hong Kong from the target population we serve in the SEED health behavioral education. They identified and ranked the indicators for measuring the following domains of children’s health: personal growth, well-being, and safety. At the time of the present impact study, some of these metrics were used to replace some version 1 of the health and health behavioral survey questions. For example, children voted as a top well-being indicator whether a child uses a positive mindset. The community scorecard study has since been completed and is yet to be prepared for publication.

### 2.4 Statistical analysis

The statistical software G*Power 3·1·9·7 sample size calculator was used to calculate the minimum sample of children required for the study as 213 (42). The sample size was calculated based on target effect size as 0·206, alpha 0·05 and power 0·85 for children’s group and for caregivers, target effect size as 0·59, alpha 0·05 and power 0·95 (44).

Descriptive statistics were reported for the sociodemographic variables (sex, age groups, ethnicity, and SEN) using means and standard deviations (SD); percentages were provided for nominal and ordinal data. Statistical analysis was conducted using SPSS (43).

Outcomes of the intervention were evaluated on four domains (health-related quality of life, emotional regulation, well-being/resilience, and safety) measured by the health behavioral survey. Paired-sample T-tests were used to test the differences between Pre-course and Post-course assessments, as well as the difference between Pre-course and Follow-Up assessments for each of the four outcome measures. Statistical significance was determined at *p* < 0.05 (two-tailed), and effect sizes were determined using Cohen’s d (0.2, small; 0.5, medium; 0.8, large).

We additionally evaluated the extent to which sociodemographic variables determined intervention outcomes measured using the health behavioral survey. As the drop-out rate at Follow-Up assessment was high, we have only modeled the difference scores between Pre-course and Post assessments for each of the four outcomes measures, with the four sociodemographic variables (sex, age groups, ethnicity, and SEN) entered simultaneously as independent variables. Linear regression models, one for each outcome measure, were used to evaluate the independent contributions of each socio-demographic variable to the Pre-Post difference scores (Table 1).

**Table 1:** Sociodemographic characteristics of children participants (*N* = 526).

Variables	n	%
Sex		
Male	338	64.3
Female	188	35.7
Age group (years)		
6–8	109	20.8
9–11	103	19.6
12–14	157	29.8
15–17	156	29.7
Ethnicity		
Chinese	416	79.1
Non-Chinese minorities	110	20.9
Special education needs (SEN)		
No	400	76
Yes	126	24

Missing data were excluded.

## 3 Results

### 3.1 Sociodemographic characteristics

526 children completed the course and the Pre-course and Post-course assessments, and 106 children completed the Follow-Up assessment. There was a larger majority of boys (64.3%) than girls (35.7%) and of Chinese children (79.1%) than non-Chinese ethnic minorities (20.9%). Among non-Chinese ethnic minority children, 61 (11.6%) identified as Pakistani, 18 (3.4%) as Indian, 13 (2.5%) as Nepalese, 4 (0.8%) as Indonesian, 3 (0.6%) as Filipino, 1 (0.2%) as Malaysian, and 10 (1.9%) as other ethnic minorities. There were comparable percentages (19.6 to 29.8%) of children across the four age groups (6–8 years, 9–11 years, 12–14 years, 15–17 years). SEN status was confirmed or suspected in 24.0% of the children.

### 3.2 Intervention outcomes measured by the health behavioral survey

Table 2 shows the outcomes of the intervention measured using the health behavioral survey at three time-points (Pre-course, Post-course, and Follow-Up). It is clear that the differences between Pre-course and Post-course assessments were statistically significant and large for each of the four outcome measures on health-related quality of life (Cohen’s d = 0.67, *p* < 0.001), emotional regulation (Cohen’s d = 0.71, *p* < 0.001), well-being/resilience (Cohen’s d = 0.66, *p < 0*.001), and safety (Cohen’s d = 0.71, *p* < 0.001). The same is true for the differences between Pre-course and Follow-Up assessments (Cohen’s d = 0.71–0.92, *p* < 0.001). Albeit with a smaller sample size, the effect sizes at Follow-Up were at least as large as that at Post-course assessment relative to the Pre-course baseline, indicating that the outcomes of the intervention were well-maintained over time.

**Table 2:** Statistical analysis of health behavioral survey across three time-points.

Variables	Time	n	Mean (SD)	T (vs. pre)	d	*p*
Health-related quality of life (*range: 11–55*)	Pre-course	526	36.7 (6.8)			
	Post-course	526	42.0 (7.4)	15.4	0.67	<0.001
	Follow-up	106	42.8 (6.2)	8.6	0.84	<0.001
Emotional regulation (*range: 5–25*)	Pre-course	526	14.5 (3.7)			
	Post-course	526	17.7 (3.9)	16.2	0.71	<0.001
	Follow-up	106	18.2 (3.0)	9.5	0.91	<0.001
Well-being/ Resilience (range: 5–25)	Pre-course	526	16.1 (3.8)			
	Post-course	526	19.0 (4.1)	15.2	0.66	<0.001
	Follow-up	106	19.1 (3.8)	7.3	0.71	<0.001
Safety (*range: 3–15*)	Pre-course	526	8.9 (2.3)			
	Post-course	526	11.0 (2.5)	16.3	0.71	<0.001
	Follow-up	106	11.3 (2.2)	9.6	0.92	<0.001

Missing data were excluded. Paired-sample T-tests were used to test the differences between Pre-course scores and Post-course scores and between Pre-course scores and Follow-Up scores. Cohen’s d for effect sizes: 0.2, small; 0.5, medium; 0.8, large.

### 3.3 Sociodemographic determinants of pre-post differences

Table 3 shows the standardized beta coefficients and their standardized 95% confidence intervals for each sociodemographic variable for each of the four outcome measures. The sociodemographic variables contributed significantly to each of the outcome measures, with each controlling for each other’s contributions. Specifically, independently of one another, the male sex was associated with greater Pre-course to Post-course increases in health-related quality of life, well-being/resilience, and safety, but not in emotional regulation. Younger age, non-Chinese ethnicity, and SEN status were associated with larger increases from Pre-course to Post-course in all four outcome measures.

**Table 3:** Linear regression models for sociodemographic determinants of pre-post differences.

Dependent variables	Independent variables	Beta	Standardized 95% C.I.
Health-related quality of life (*Post-Pre*)	Sex (Female = 1)	−0.15	(−0.23, −0.07)
	Age groups	−0.22	(−0.30, −0.13)
	Ethnicity (Non-Chinese = 1)	0.11	(0.02, 0.19)
	SEN (Yes = 1)	0.18	(0.09, 0.26)
Emotional regulation (*Post-Pre*)	Sex (Female = 1)	−0.06	(−0.14, 0.02)
	Age groups	−0.26	(−0.34, −0.18)
	Ethnicity (Non-Chinese = 1)	0.16	(0.08, 0.24)
	SEN (Yes = 1)	0.21	(0.13, 0.29)
Well-being/ Resilience (*Post-Pre*)	Sex (Female = 1)	−0.13	(−0.21, −0.04)
	Age groups	−0.23	(−0.31, −0.15)
	Ethnicity (Non-Chinese = 1)	0.18	(0.10, 0.26)
	SEN (Yes = 1)	0.2	(0.12, 0.28)
Safety (*Post-Pre*)	Sex (Female = 1)	−0.1	(−0.18, −0.02)
	Age groups	−0.16	(−0.25, −0.08)
	Ethnicity (Non-Chinese = 1)	0.17	(0.09, 0.26)
	SEN (Yes = 1)	0.16	(0.08, 0.25)

Dependent variables were the differences between the pre-course scores and post-course scores of the health behavioral survey. Follow-up scores were omitted. Separate linear regression models were used for each outcome measure. All independent sociodemographic variables were entered into the models simultaneously.

To further delineate the source of these sociodemographic effects, we further analyzed the Pre-course and Post-course scores separately to determine if there were sociodemographic differences at baseline before the intervention (Pre-course) and if any of these differences changed immediately after intervention (Post-course). Using linear regression models (Supplementary Table 1), we found that, at the Pre-course baseline, the male sex was associated with lower well-being/resilience. Older age was associated with lower health-related quality of life and well-being/resilience. Non-Chinese ethnic minority status was associated with lower health-related quality of life, emotional regulation, and well-being/resilience. SEN status was associated with lower health-related quality of life, emotional regulation, well-being/resilience, and safety. Thus, there was evidence of sociodemographic disparities in health, well-being, and safety at Pre-course baseline; those of older age, non-Chinese ethnic minorities, and SEN children were systematically more disadvantaged before the intervention.

Using linear regression models at Post-course assessment (Supplementary Table 2), we found that the male sex was associated with higher health-related quality of life and safety. Older age was associated with lower health-related quality of life, emotional regulation, well-being/resilience, and safety (to a greater extent compared with Pre-course baseline). At Post-course assessment, non-Chinese ethnic minority status was associated with *higher* emotional regulation, well-being/resilience, and safety; SEN status was without effects Post-course. Thus, the disproportionate lower baseline levels of health of being in the non-Chinese ethnic minority or SEN status was reversed or eliminated after the intervention. Age disparities appeared to have increased, favoring younger children both Pre-and Post-course, as well as larger positive changes from Pre-course to Post-course assessment (see Table 3). Sex differences appeared less systematic, except for greater positive changes for boys than girls from Pre-to Post-course (see Table 3).

### 3.4 Comparison among three different class environments

Of the 526 children, 296 (56.3%) participated in the SEED at their school, 136 (25.9%) at our community center, and 94 (17.9%) at the other non-profit organization where they enrolled. Community course participants’ caregivers signed up to participate in the Be Priceless center through the organization’s website. In both the school and non-profit organization, their staff oversaw the course promotion, recruitment, and coordination. All courses were taught by SEED Educators and had the same content, structure, and approximate duration.

Using logistical regression, we found that the environment also had no significant association changes in Pre-course to Post-course assessment (Beta −0.03, 95% CI −0.12 to 0.06) and Pre-course to Follow-up assessment (Beta 0.14, 95% CI −0.14 to 0.53).

Out of the 106 children who completed the follow-up survey, 84 (77.4%) were from school, 18 (17%) were from community, and 7 (6.6%) were from other non-profit organizations. Follow-up total survey score, we found that the environment that classes were conducted in had no significant association (Beta 0.51, 95% CI −0.26 to 0.41).

## 4 Discussion

The present study used a pre-post intervention design to evaluate the outcomes of our health-behavioral educational intervention on children aged 6 to 17. On all four domains of our outcome measures, namely, health-related quality of life, emotional regulation, well-being/resilience, and safety, we found significant increases from Pre-course to Post-course as well as over a follow-up period of about 6 months (among those we followed-up), with comparably large effect sizes relative to baseline at both time-points after the intervention.

At baseline, there were significant sociodemographic disparities among our children participants. We observed that older children, non-Chinese ethnic minorities, and SEN children were more disadvantaged in terms of their health, well-being, and safety even before the intervention. This provides evidence of health inequities among Hong Kong children in our community. Critically, immediately after intervention, the ethnic and SEN disparities observed at baseline were reversed or eliminated. This suggests that our intervention helped bridge the difference between advantaged and disadvantaged children. This interpretation is further strengthened by our finding that the pre-course to Post-course increases in our outcome measures were greater for non-Chinese ethnic minorities and SEN children relative to their Chinese and non-SEN counterparts. Thus, our intervention program evidently promoted health equity and inclusivity for Hong Kong children in our community.

On the other hand, the age disparities that existed before the intervention (favoring younger over older children in terms of their health, well-being, and safety) appeared to have increased further after the intervention. This is likely attributable to the greater increases in all four of the outcome measures from Pre-course to Post-course. These findings suggest that earlier intervention could lead to greater benefits among our children participants. They also show that it is not too late to improve outcomes of older children, even in the latter stage of secondary school. Children aged 6 to 17 differed in their physical-psychosocial developments and needs. It is a period of marked social and emotional changes as they develop across puberty and adolescence, as well as substantial educational attainments as they graduate from primary to secondary school. The finding that younger children appeared to benefit more from our intervention suggests that more age-appropriate materials should be developed for older children, for example addressing growing needs to develop self-identity, impulse control, and interpersonal safety. This remains to be done in our future interventions and research.

### 4.1 Strengths and limitations

Of the 629 number of children who started the course 526 (83.6%) completed it. There is a chance that those who completed the course had higher motivation and hence had greater influence on the results. Out of the children who completed the course, only 106 (20.2%) were followed up post-course due to our resource limitations for conducting extensive follow-up. We prioritized following up the sustained benefit for at-risk children for health equity reasons. Of those who were followed-up post-course, 80% were from low-income, non-Chinese ethnic minorities or SEN contexts. The incomplete follow-up of all children limits the generalizability of the findings to all children, especially to those who are not from the at-risk groups.

There are several strengths of this study. This describes an effective intervention tailored for at-risk children’s mental, physical, and social health. The comprehensive course integrates evidence-based approaches from diverse fields and is a behavioral intervention that aims to improve children’s mental, physical, and social health, well-being and safety. The impact measurement survey was developed by a trans-sectoral community that includes children, caregivers, and professionals with independent expertise in evaluation or research from outside the program. The sample size is substantial, supporting the results of this study.

## 5 Conclusion

In conclusion, we found large increases in health, emotional regulation, well-being, and safety among our children participants immediately after our health-behavioral intervention, and, for those we followed-up, these increases were well-maintained across a period of about 6 months. We also found evidence that our intervention has reduced ethnic and SEN disparities, thereby promoting health equity and inclusivity for marginalized children in the Hong Kong community. Further work should be done to develop more age-appropriate materials for our intervention and research that promotes comparable benefits for children of different ages.

## Data Availability

The raw data supporting the conclusions of this article will be made available by the authors, without undue reservation.
