# Supplementary material for: Improving children and adolescents’ quality of life, personal growth, well-being, and safety through health-behavioral education: a pre-post intervention study
Source: Front Public Health. 2025 Apr 17;13:1527268. doi: 10.3389/fpubh.2025.1527268 (PMC12043601; doi:10.3389/fpubh.2025.1527268)
Supplement: Supplementary file 1 [file Table_1.docx]

**Supplementary Table 1**. Linear regression models for sociodemographic determinants of Pre-course scores

| **Dependent variables** | **Independent variables** | **Beta** | **Standardized**  **95% C.I.** |
| --- | --- | --- | --- |
| Health-related quality of life  *(Pre-course)*  Emotional regulation  *(Pre-course)*  Well-being/ Resilience  *(Pre-course)*  Safety  *(Pre-course)* | Sex (Female = 1)  Age groups  Ethnicity (Non-Chinese = 1)  SEN (Yes = 1)  Sex (Female = 1)  Age groups  Ethnicity (Non-Chinese = 1)  SEN (Yes = 1)  Sex (Female = 1)  Age groups  Ethnicity (Non-Chinese = 1)  SEN (Yes = 1)  Sex (Female = 1)  Age groups  Ethnicity (Non-Chinese = 1)  SEN (Yes = 1) | 0.07  -0.11  -0.09  -0.16  -0.01  0.05  -0.10  -0.17  0.17  -0.12  -0.10  -0.20  0.06  -0.07  -0.05  -0.15 | (-0.02, 0.16)  (-0.20, -0.02)  (-0.17, -0.01)  (-0.25, -0.08)  (-0.10, 0.07)  (-0.04, 0.14)  (-0.19, -0.02)  (-0.26, -0.09)  (0.09, 0.26)  (-0.21, -0.04)  (-0.18, -0.02)  (-0.29, -0.12)  (-0.03, 0.14)  (-0.16, 0.02)  (-0.14, 0.04)  (-0.24, -0.07) |

*Note*. Dependent variables were the Pre-course scores of the health behavioral survey. Separate linear regression models were used for each outcome measure. All independent sociodemographic variables were entered into the models simultaneously.
