# Supplementary material for: Improving children and adolescents’ quality of life, personal growth, well-being, and safety through health-behavioral education: a pre-post intervention study
Source: Front Public Health. 2025 Apr 17;13:1527268. doi: 10.3389/fpubh.2025.1527268 (PMC12043601; doi:10.3389/fpubh.2025.1527268)
Supplement: Supplementary file 2 [file Table_2.docx]

**Supplementary Table 2**. Linear regression models for sociodemographic determinants of Post-course assessment scores

| **Dependent variables** | **Independent variables** | **Beta** | **Standardized**  **95% C.I.** |
| --- | --- | --- | --- |
| Health-related quality of life  *(Post-course)*  Emotional regulation  *(Post-course)*  Well-being/ Resilience  *(Post-course*)  Safety  *(Post-course)* | Sex (Female = 1)  Age groups  Ethnicity (Non-Chinese = 1)  SEN (Yes = 1)  Sex (Female = 1)  Age groups  Ethnicity (Non-Chinese = 1)  SEN (Yes = 1)  Sex (Female = 1)  Age groups  Ethnicity (Non-Chinese = 1)  SEN (Yes = 1)  Sex (Female = 1)  Age groups  Ethnicity (Non-Chinese = 1)  SEN (Yes = 1) | -0.10  -0.33  0.03  0.04  -0.08  -0.25  0.09  0.08  0.02  -0.36  0.11  0.03  -0.06  -0.26  0.16  0.05 | (-0.18, -0.02)  (-0.42, -0.25)  (-0.05, 0.12)  (-0.04, 0.13)  (-0.16, 0.01)  (-0.34, -0.17)  (0.002, 0.17)  (-0.002, 0.16)  (-0.06, 0.11)  (-0.44, -0.28)  (0.03, 0.19)  (-0.05, 0.11)  (-0.15, -0.02)  (-0.34, -0.17)  (0.08, 0.24)  (-0.03, 0.14) |

*Note*. Dependent variables were the Post-course scores of the health behavioral survey. Separate linear regression models were used for each outcome measure. All independent sociodemographic variables were entered into the models simultaneously.
